# Resposta à Carta ao Editor: "O Treinamento Resistido Também Deve Ser Considerado um Componente Obrigatório?"

**DOI:** 10.36660/abc.20260362

**Published:** 2026-07-02

**Authors:** Cibele Isaac Saad Rodrigues, Claudia Lucia de Moraes Forjaz, Luiz Aparecido Bortolotto, Wilson Nadruz, Andréa A. Brandão

**Affiliations:** 1 Pontifícia Universidade Católica de São Paulo Faculdade de Ciências Médicas e da Saúde São Paulo SP Brasil Pontifícia Universidade Católica de São Paulo – Faculdade de Ciências Médicas e da Saúde, São Paulo, SP – Brasil; 2 Escola de Educação Física e Esporte – Universidade de São Paulo São Paulo SP Brasil Escola de Educação Física e Esporte – Universidade de São Paulo, São Paulo, SP – Brasil; 3 Universidade de São Paulo Instituto do Coração do Hospital das Clínicas Faculdade de Medicina São Paulo SP Brasil Instituto do Coração do Hospital das Clínicas da Faculdade de Medicina da Universidade de São Paulo, São Paulo, SP – Brasil; 4 Universidade Estadual de Campinas Campinas SP Brasil Universidade Estadual de Campinas (UNICAMP), Campinas, SP – Brasil; 5 Universidade do Estado do Rio de Janeiro Rio de Janeiro RJ Brasil Universidade do Estado do Rio de Janeiro, Rio de Janeiro, RJ – Brasil

**Keywords:** Hipertensão, Treinamento Resistido, Condicionamento Físico Humano, Treino Aeróbico, Guia de Prática Clínica

O treinamento resistido (TR) é, sem dúvida, um componente essencial de um programa de exercícios abrangente para melhorar e manter o condicionamento físico, a saúde global e a qualidade de vida. Exercícios aeróbicos, de resistência e de alongamento, realizados em conjunto regularmente, promovem benefícios fisiológicos, motores, cognitivos e emocionais significativos, sendo recomendados para adultos e idosos.^1,2^

Embora, tanto o treinamento aeróbico (TA) quanto o TR tenham impactos em diferentes dimensões da saúde, há evidências robustas e de alta qualidade de que o TR é a modalidade de treinamento mais eficiente para melhorar a estrutura e a função muscular, sendo obrigatório para a prevenção e o tratamento de doenças musculoesqueléticas, o que o torna particularmente importante para idosos.^1-4^ Por outro lado, as evidências sobre os efeitos do TR em parâmetros e doenças cardiovasculares são mais recentes e menos robustas.

Especificamente em relação à hipertensão, uma revisão guarda-chuva de Morita et al.^5^ concluiu que o TR foi tão eficaz quanto o TA para reduzir a pressão arterial em pacientes com hipertensão. Esse achado, no entanto, não é consistente na literatura. Uma meta-revisão^6^ conduzida pela Associação Europeia de Cardiologia Preventiva e pelo Conselho de Hipertensão da Sociedade Europeia de Cardiologia concluiu que pacientes com hipertensão parecem se beneficiar mais do TA. O editorial do periódico em que a revisão de Morita et al. foi publicada^7^ apontou diversas limitações em relação à conclusão da revisão. Os resultados dos estudos incluídos na revisão apresentaram heterogeneidade extremamente alta (I^2^ = 99,1%) e houve uma clara assimetria nos gráficos de funil. Em conjunto, esses aspectos mostraram alta variabilidade na resposta da pressão arterial ao TR, sugerindo viés de publicação contra resultados nulos. Portanto, parece imprecisa a generalização de que o TR e o TA são igualmente eficazes no tratamento da hipertensão em todos os subgrupos de pacientes e para qualquer protocolo de TR.

Outro aspecto importante é o fato de que a maioria dos estudos conduzidos com TR e pacientes com hipertensão avaliou a pressão arterial aferida no consultório.^7^ É bem conhecido que a pressão arterial ambulatorial de 24 horas se associa mais fortemente a danos em órgãos mediados pela hipertensão e à morbimortalidade cardiovascular do que a pressão arterial aferida no consultório.^8-10^ A pressão arterial ambulatorial de 24 horas também é considerada o método mais confiável para monitorar os efeitos do tratamento anti-hipertensivo.^8,11^ Uma metanálise que avaliou especificamente os efeitos do treinamento na pressão arterial ambulatorial de 24 horas encontrou apenas três ensaios clínicos randomizados controlados de boa qualidade com TR dinâmico e concluiu que o TR não alterou a pressão arterial ambulatorial de 24 horas em pacientes com hipertensão.^12^

Finalmente, os estudos sobre TR são principalmente de curta duração, não permitindo a avaliação da adesão ao tratamento e da sustentabilidade do efeito hipotensor, que são essenciais para o sucesso do tratamento.^7^ Além disso, a segurança do TR não foi suficientemente estudada, especialmente para subgrupos de pacientes com hipertensão que podem apresentar lesão de órgãos-alvo, risco de aneurisma e outros fatores de risco. Mais uma vez, parece inadequada uma recomendação geral de TR na hipertensão sem conhecimento consistente sobre esses aspectos.

Portanto, com base nas limitações das revisões, na falta de efeito comprovado do TR na pressão arterial ambulatorial de 24 horas, na escassa literatura sobre seus possíveis efeitos adversos e manutenção dos benefícios a longo prazo, as evidências científicas foram consideradas insuficientes para incluir o TR como terapia obrigatória na hipertensão. No entanto, os importantes benefícios do TR para a saúde global e a qualidade de vida foram reconhecidos na Diretriz Brasileira de Hipertensão Arterial – 2025, que recomenda TA complementada por TR para pacientes com hipertensão (Tabela 6.3),^13^ bem como fornece recomendações para programas de TR na hipertensão.

Em conclusão, em relação à pergunta "O treinamento resistido também deve ser considerado um componente obrigatório?", para hipertensão, a resposta é "ainda não".
