# Supplementary material for: Resposta à Carta ao Editor: "O Treinamento Resistido Também Deve Ser Considerado um Componente Obrigatório?"
Source: Arq Bras Cardiol. 2026 Jul 2;123(6):e20260362. [Article in Portuguese] doi: 10.36660/abc.20260362 (PMC13399065; doi:10.36660/abc.20260362)
Supplement: Translation

# Translation: Reply to the Letter to the Editor: "Should Resistance Training Also Be Considered a Mandatory Component?"

**DOI:** 10.36660/abc.20260362i

**Authors:** Cibele Isaac Saad Rodrigues, Claudia Lucia de Moraes Forjaz, Luiz Aparecido Bortolotto, Wilson Nadruz, Andréa A. Brandão

**Affiliations:** 1 São Paulo SP Brazil Pontifícia Universidade Católica de São Paulo – Faculdade de Ciências Médicas e da Saúde, São Paulo, SP – Brazil; 2 São Paulo SP Brazil Escola de Educação Física e Esporte – Universidade de São Paulo, São Paulo, SP – Brazil; 3 São Paulo SP Brazil Instituto do Coração do Hospital das Clínicas da Faculdade de Medicina da Universidade de São Paulo, São Paulo, SP – Brazil; 4 Campinas SP Brazil Universidade Estadual de Campinas (UNICAMP), Campinas, SP – Brazil; 5 Rio de Janeiro RJ Brazil Universidade do Estado do Rio de Janeiro, Rio de Janeiro, RJ – Brazil

**Keywords:** Hypertension, Resistance Training, Physical Conditioning Human, Endurance Training, Practice Guideline

Resistance training (RT) is undoubtedly an essential component of a comprehensive exercise program for improving and maintaining physical fitness, global health, and quality of life. Aerobic, resistance, and stretching exercises performed together on a regular basis promote significant physiological, motor, cognitive, and emotional benefits and are recommended for adults and older adults.^1,2^

Although both aerobic training (AT) and RT have impacts on different health dimensions, there is high quality, robust evidence that RT is the most efficient mode of training for improving muscle structure and function, and it is mandatory for prevention and treatment of musculoskeletal diseases, making it particularly important for older adults.^1-4^ On the other hand, evidence about the effects of RT on cardiovascular parameters and diseases is more recent and less robust.

Specifically concerning hypertension, a recent umbrella review from Morita et al.^5^ concluded that "RT was as effective as aerobic training for reducing blood pressure in hypertension." This finding, however, is not consistent in the literature. A meta-review^6^ conducted by the European Association of Preventive Cardiology and the ESC Council on Hypertension concluded that "Patients with hypertension seem to benefit most from AT." The editorial of the journal in which the review by Morita et al. was published^7^ pointed out several limitations regarding the review's conclusion. The results of the studies included in their review presented extremely high heterogeneity (I^2^ = 99.1%), and there was a clear asymmetry in the funnel plots. Together, these aspects show high variability in blood pressure response to RT and suggest publication bias against null results. Therefore, the generalization that RT and AT are equally effective in hypertension treatment across all patient subsets and RT protocols seems imprecise.

Another important aspect is the fact that most of the studies conducted with RT and patients with hypertension have evaluated office blood pressure.^7^ It is well known that 24-hour ambulatory blood pressure is more strongly associated with hypertension-mediated organ damage and cardiovascular morbidity and mortality than office blood pressure.^8-10^ Additionally, 24-hour ambulatory blood pressure is considered the most reliable method for monitoring the effects of anti-hypertensive treatment.^8,11^ A meta-analysis that specifically evaluated the training effects on 24-hour ambulatory blood pressure found only three good quality randomized controlled trials with dynamic RT and concluded that RT did not change 24-hour blood pressure in hypertension.^12^

Finally, studies regarding RT are mainly of short duration, not allowing evaluation of adherence to treatment and sustainability of the blood pressure lowering effect, which are essential for successful treatment.^7^ In addition, safety of RT has not been sufficiently studied, especially for subsets of patients with hypertension who may present target-organ damage, risk of aneurysm, and other risk factors. Once again, a general recommendation of RT in hypertension without consistent knowledge regarding these aspects seems inappropriate.

Therefore, based on the limitations of the reviews, the lack of proven effect of RT on 24-hour ambulatory blood pressure, the scarce literature regarding its possible adverse effects and long-term maintenance of benefits, the scientific evidence was considered insufficient to include RT as a mandatory therapy in hypertension. Nevertheless, the important benefits of RT for global health and quality of life were recognized in the Brazilian Guidelines of Hypertension – 2025, which recommend AT complemented by RT for patients with hypertension (Table 6.3)^13^ and also provide recommendations for RT programs in hypertension.

In conclusion, regarding the question "Should resistance training also be considered a mandatory component?", for hypertension, the answer is "not yet."
